# Diabetes foot disease: the Cinderella of Australian diabetes management?

**DOI:** 10.1186/1757-1146-5-24

**Published:** 2012-10-01

**Authors:** Peter A Lazzarini, Joel M Gurr, Joseph R Rogers, Andrew Schox, Shan M Bergin

**Affiliations:** 1 Allied Health Research Collaborative, Metro North Health Service District, Queensland Health, Brisbane, Australia; 2 School of Clinical Sciences, Queensland University of Technology, Brisbane, Australia; 3 Podiatry Department, Royal Perth Hospital, South Metropolitan Health Service, WA Health, Perth, Australia; 4 John Morris Diabetes Centre, Launceston General Hospital, Launceston, Australia; 5 The Perth Foot and Ankle Clinic, Perth, Australia; 6 Diabetic Foot Unit and High Risk Foot Service, Dandenong Hospital, Southern Health, Victoria, Australia

**Keywords:** Diabetes, Foot, Complication, Disease, Australia

## Abstract

Diabetes is one of the greatest public health challenges to face Australia. It is already Australia’s leading cause of kidney failure, blindness (in those under 60 years) and lower limb amputation, and causes significant cardiovascular disease. Australia’s diabetes amputation rate is one of the worst in the developed world, and appears to have significantly increased in the last decade, whereas some other diabetes complication rates appear to have decreased. This paper aims to compare the national burden of disease for the four major diabetes-related complications and the availability of government funding to combat these complications, in order to determine where diabetes foot disease ranks in Australia. Our review of relevant national literature indicates foot disease ranks second overall in burden of disease and last in evidenced-based government funding to combat these diabetes complications. This suggests public funding to address foot disease in Australia is disproportionately low when compared to funding dedicated to other diabetes complications. There is ample evidence that appropriate government funding of evidence-based care improves all diabetes complication outcomes and reduces overall costs. Numerous diverse Australian peak bodies have now recommended similar diabetes foot evidence-based strategies that have reduced diabetes amputation rates and associated costs in other developed nations. It would seem intuitive that “it’s time” to fund these evidence-based strategies for diabetes foot disease in Australia as well.

## Background

Diabetes has been coined the ‘Black Death of the 21^st^ century’ due to its stark similarities with the 14^th^ century plague in terms of the rapid increase in global prevalence, morbidity and mortality [1]. In 2004, the global forecast of people with diabetes was estimated at 366 million by 2030 [2]. Alarmingly this figure (equivalent to 8.3% of the world’s adult population) was reached in 2011 almost 20 years earlier than expected [3]. The updated 2030 estimate is now a staggering 552 million people (10% of the world’s adult population) [3]. These disturbing figures prompted the United Nations to convene only the second ever UN General Assembly summit on a health issue in 2011 [4]. The summit resulted in 193 nations unanimously agreeing to adopt a Political Declaration on diabetes and other non-communicable diseases that commits them to take action to urgently stem the tide of diabetes and its consequences [4,5].

Diabetes is characterised by high blood glucose levels, which can result in significant morbidity and mortality from macrovascular and microvascular complications including heart attack, stroke, kidney failure, blindness and lower limb amputation [5-7]. The significance of the complications faced by people with diabetes is emphasised by the fact that someone in the world dies from diabetes-related complications every seven seconds [5] and diabetes is now the leading cause of kidney failure and lower limb amputations globally [7]. In alignment with the overall increase in diabetes prevalence, healthcare expenditure on diabetes is expected to rise significantly from $US465 billion in 2011 to $US595 billion in 2030 [5]. This has prompted the World Economic Forum to identify diabetes as a global risk for business [5].

Australia is far from immune from the increasing impact of diabetes, with experts suggesting that it presents one of the most significant public health challenges Australia has ever faced [7,8]. Australia currently has over 1 million people diagnosed with diabetes [9], representing 7.4% of the adult population [6]. An estimated additional 500,000 Australians have diabetes but remain undiagnosed [7]. By 2025, it is estimated that 2.5 to 3 million Australians will have diabetes [10,11]. This is equivalent to one in every three adults developing diabetes in their lifetime. [10,11]. The consequences of diabetes in Australia are significant with over 500,000 hospital admissions (8% of all admissions) and 12,000 deaths (9% of all deaths) attributable to the condition in 2004 alone [6]. Consistent with global reports diabetes is the leading cause of kidney disease and lower limb amputation in Australia [7,12], resulting in increased length of hospital stay [6] and a cost of approximately $A6 billion in 2003 [13].

Successive Australian Governments have not ignored the ‘silent pandemic’ of diabetes. Diabetes was announced as the fifth National Health Priority Action (NHPA) area in 1996 and as such governments have invested in strategic plans, best-practice guidelines, medications, consumables, service programs, research and educational campaigns in an attempt to optimise care and reduce the impact of related complications [14,15]. The first *National Diabetes Strategic Plan* (1998) identified over 60 recommendations aimed at reducing the prevalence of diabetes and its complications [15]. This strategic plan strongly urged implementing a suite of nationally coordinated priority programs aimed at tackling individual diabetes complications in particular [15]. These proposed national programs included a visual impairment prevention program, end stage renal disease prevention program, cardiovascular disease prevention program and a foot disease management program [15]. The plan went further and expressly outlined it expected a 50% reduction in all diabetes-related end-stage complications within a decade with the implementation of all programs [15].

Despite these best intentions, a recent paper published in the *Medical Journal of Australia* by the Australian Diabetes Society has suggested that Australia’s diabetes-related amputation numbers have increased by 30% between 1998 and 2005 and that Australia has failed to successfully implement key best-practice recommendations outlined for the management of diabetes-related foot disease [16]. More recent data suggests that diabetes-related amputation rates (per 100,000 general population) have also increased in Australia by over 30% for the period between 1998 and 2011 [17-19]. By comparison a recently released national diabetes indicator report shows a 30% reduction in vision loss over a similar period [17]. Furthermore, the same report indicates that new rates of end stage foot complications (amputations) are up to four times higher than that of new end-stage kidney disease [17].

The apparent disparity between rates and outcomes for foot disease compared to other diabetes-related complications prompted the authors to consider whether this correlates to an inequality in the level of government funding foot disease receives. Thus, this paper aims to firstly compare the national burden of disease for different diabetes-related complications and secondly the availability of government funding to combat these complications, in order to determine specifically where diabetes-related foot disease ranks in terms of disease burden and government spending.

## National burden and funding of diabetes complications

Successive Australian governments have overseen the implementation of multi-faceted strategies in an attempt to turn the tide of diabetes over the last fifteen years. The National Health & Medical Research Council (NHMRC) has taken the lead on the development of best-practice guidelines and recommendations designed to encourage health systems and professionals to improve the management of diabetes and its complications [20-23]. Australian governments have attempted to complement these guidelines by funding best-practice diabetes management recommendations via its Medical Benefits Schedule (MBS), Pharmaceutical Benefits Scheme (PBS) and other nationally funded programs aimed at tackling diabetes complications. With the release of multiple national government data reports [6,17] and successive research publications over the last decade [24-32], it is timely to compare the impact of these strategies on diabetes complication rates, costs, burden of disease and public health funding in Australia.

A review of relevant national diabetes complication-related literature published over the last 15 years forms the basis of this paper. Publications reviewed included government reports, epidemiological publications, health economic publications, evidence-based guidelines, government media releases, Medicare and Pharmaceutical Benefits Schemes.

Table 1 displays the prevalence rates of diabetes-related high-risk complications, history of acute end stage complications (“acute complications”), new annual incidence of acute end stage complications, hospital admissions, average length of stay, deaths and burden of disease found in people with diabetes in Australia. For the purposes of this paper, we have defined ‘high-risk complications’ as those diabetes-related complications (e.g. peripheral neuropathy, chronic kidney disease, retinopathy or heart failure) that place a person at high-risk of developing a more serious acute end stage diabetes complication (i.e. amputation, end stage renal disease, blindness or myocardial infarction respectively).

**Table 1:** Comparison of specific adult diabetes complication rates, hospitalisations, deaths and burden of disease in Australia

	**High-risk**^**a**^		**Acute complications**			**Hospitalisation**		**Burden of disease**	
	Population*	Service**	**History - population**^b*^	**History – service**^c**^[25]	**New –Service**^c**^[25]	**Admissions**^**d**^[6]	**ALOS**^**d**^[6]	**Deaths**^**d**^[6]	**Burden Dx**^**e**^**(DALY)**^**f**^[6]
**CVD**	21% [31]	40% [32]	NA	14.0%	4.3%	93,110	NA	7,617	74,687
**Kidney**	16% [30]	28% [25]	0.3% [30]	1.5%	0.3%	102,000	9	1,557	2,483
**Eyes**	15% [28]	29% [25]	2.1% [28]	0.9%	0.7%	38,700	NA	NA	1,258
**Foot**	20% [27]	25% [25]	2.1% [27]	1.5%	0.6%	59,240	12-26	1,705	18,544

ALOS: average length of stay; CVD: cardiovascular disease; DALY: disability adjusted life years; NA: not available.

*Population: Figures are taken from a population based study.

**Service: Figures are taken from a service-based study.

^a^ High Risk: CVD (coronary heart disease, cerebrovascular disease, peripheral vascular disease), Kidney (chronic kidney disease stage 3, eGFR < 60 ml/min per 1.73 m^2^), Eyes (retinopathy), Foot (peripheral neuropathy, peripheral arterial disease).

^b^ Acute Complication History – Population based: Kidney (chronic kidney disease stage 4 & 5, eGFR < 30 ml/min per 1.73 m^2^), Eyes (proliferative diabetes retinopathy), Foot (foot ulcer).

^c^ Acute Complication History – Service based: CVD (myocardial infarct, cerebral stroke), Kidney (end stage renal disease, eGFR < 30 ml/min per 1.73 m^2^), Eyes (blindness), Foot (lower limb amputation).

^d^ Hospitalisation: CVD (coronary heart disease, stroke), Kidney (chronic kidney failure), Eyes (retinopathy, glaucoma, cataract), Foot (peripheral neuropathy, peripheral vascular disease, foot ulcer, lower limb amputation).

^e^ Burden of disease: CVD (ischaemic heart disease, stroke), Kidney (renal failure), Eyes (retinopathy), Foot (peripheral neuropathy, peripheral vascular disease, foot ulcer, lower limb amputation).

^f^ DALY: Healthy life years lost in terms of illness, injury and premature death. DALY combines years of life lost to premature death and years of healthy life lost due to non-fatal health outcomes.

### How many people are affected by the different diabetes complications in Australia?

Diabetes contributes significantly to cardiovascular, kidney, eye and foot disease [5,7]. Alarmingly, diabetes is now Australia’s leading cause of kidney failure, blindness (in people under 60 years) [7] and lower limb amputation [12]; accounting for 17% of all blindness, 32% of all end stage kidney failure and 60% of all amputations [6,12]. Recent reports suggest Australia has one of the worst diabetes-related lower limb amputation rates in the developed world [12,33,34], at nearly 20 per 100,000 people with diabetes [17,18,34] compared to an average of 12 per 100,000 people with diabetes in the developed world [33].

Our analysis suggest that foot disease ranks second overall to cardiovascular disease in terms of numbers of people affected by a high-risk or acute complication in the Australian population with diabetes (Table 1). Further figures suggest new acute complication cases are approximately 18 per 100,000 people per year for amputation and blindness, compared to six per 100,000 per year for end stage renal disease [17].

### How much health care resource do different diabetes complications use in Australia?

Foot disease ranks third behind cardiovascular and kidney disease in terms of the numbers of diabetes-related acute hospital admissions (Table 1) [6]. However, diabetes-related foot disease results in longer average length of hospital stay when compared to all other diabetes-related complications (Table 1) [6]. Thus, it could be argued that in Australia diabetes-related foot complications consume the second largest amount of acute care resources of all diabetes complications behind only kidney disease when total hospital bed day usage is taken into account. A 2008 Australian study supports the contention that diabetes-related foot disease consumes the highest number of bed days annually, reporting 48 and 57 days respectively for admitted foot ulcers and amputations (compared to 18 days for myocardial infarction for example) [35]. Furthermore, the same study suggested that annual primary care resources (i.e. GP visits only) required to manage a diabetes complication for the year following an acute episode was highest for an acute foot complication; requiring an average of 18 GP visits for a foot ulcer [35].

### How much do different acute diabetes complications cost in Australia?

It has been reported that a person with diabetes (type 1 or type 2) without complications costs Australia around $3,500 to $4,000 per year in healthcare costs, and this escalates to around $7,000 for people with microvascular complications, and $16,700 per year in people with microvascular and macrovascular complications [13,36]. Acute foot complications (foot ulcers and amputations) are reported as having both a micro- and macrovascular component [13].

Table 2 displays costs associated with different diabetes-related complications broken down into hospital costs and non-hospital costs (medical and pharmaceutical costs only) for the initial year following a new acute complication and for each subsequent year after that in Australia. The literature suggests that amputations and foot ulcers are consistently the second and third most expensive acute diabetes complications respectively to treat in terms of both hospital and out-of-hospital costs [35]. Renal failure is by far the most expensive acute care complication and consumes further hospital resources via dialysis management [35,37].

**Table 2:** Comparison of specific costs for first diabetes complication episodes in Australia

		**Initial year of complication event**				**Each subsequent year of complication event**		
		**Total $A 2003**[37]*	**Total $A 1999**[35]**	**Hospital $A 1999**[35]**	**Non-hospital $A 1999**[35]**	**Total $A 1999**[35]**	**Hospital $A 1999**[35]**	**Non-hospital $A 1999**[35]**
**CVD**	**MI**	$15,611	$11,660	$10,836	$824	$2,701	$2,036	$665
	**CVA**	$17,073	$14,012	$13,032	$980	$3,691	$2,924	$767
**Kidney**	**ESRF**	$34,991	$28,661	$27,820	$841	$30,612	$29,952	$660
**Eyes**	**Blind**	$26,548	$8,880	$8,126	$754	$3,039	$2,425	$615
**Foot**	**Amputations**	$23,555	$23,793	$22,991	$802	$6,065	$5,313	$751
	**Foot ulcer**	$14,691	$18,246	$17,357	$889	$4,761	$4,077	$684

* Data converted from Euro to $A at 2012 rates (1 Euro = $1.27).

** Note cost is the higher estimate from either a 60yo male or a 70yo male.

### What is the burden of disease of the different diabetes complications in Australia?

Australian burden of disease and mortality statistics (Table 1) demonstrate that foot complications rank second only to cardiovascular disease in terms of the effect all diabetes complications have on lost healthy years suffered by the Australian community [6]. Burden of disease can be summarised by disability adjusted life years (DALY) figures that determine total lost healthy life years in terms illness, injury and premature death [6]. Furthermore, mortality data associated with diabetes complications suggests foot complications are the second leading cause of diabetes-related death second only to cardiovascular disease; cardiovascular disease accounts for four-times the rate of any other diabetes cause [6].

### How does the Australian government fund best-practice management of different diabetes complications?

The Australian Government and NHMRC have regularly commissioned best-practice guidelines for the management of various diabetes complications [20-23]. Each guideline formulates recommendations (NHMRC graded) using standard evidence-based methods and are endorsed by NHMRC [20-23]. There are broad similarities in the recommendations emanating from the suite of diabetes complication guidelines including; screening every one to two years for complications, control of blood glucose levels, blood pressure and lipids, requirements for further investigations and/or surgical intervention for acute complications and a multi-disciplinary approach [20-23].

The cost of most of the NHMRC endorsed diabetes complication guideline recommendations are funded via the Australian Government’s MBS or PBS programs [38,39]. The MBS Program, under the auspices of the *Health Insurance Act 1973* (Cth) provides access to services via Medicare benefits which are claimable only for ‘clinically relevant’ services provided by an appropriate health practitioner [38]. The PBS program, through the *National Health Act 1953* (Cth), “provides timely, reliable and affordable access to necessary medicines for Australians” [39].

An examination of the best-practice guideline recommendations endorsed by the NHMRC for both assessment and management of diabetes and its complications reveals that all recommended *assessments* are publicly funded by the MBS scheme [20-23,38]. Interestingly, in terms of *management* of diabetes complications, it appears that all but possibly one of the recommendations outlined for cardiovascular, kidney or eye disease are funded via the MBS; the exception appears to be intravitreal triamcinolone for persistent diabetes macular degeneration management [20-23,38]. In contrast, it appears that at least five evidence-based recommendations outlined in the NHRMC diabetes guidelines to manage foot complications are not funded by the MBS [23,38]. These include:

(i) total contact cast or other device rendered irremovable;

(ii) topical hydrogel and wound dressings;

(iii) appropriate footwear;

(iv) topical negative pressure therapy; and

(v) larval therapy for complex acute foot complication management [23,38].

Furthermore, it should be noted that the MBS schedule for podiatry consultation is capped at a maximum of five allied health consultations rebatable annually, and therefore, podiatry ‘competes’ with all other allied health consultations [38]. Given that two other diabetes foot guideline recommendations specifically include the necessity for multiple podiatry review consultations in high-risk diabetes foot complication management and diabetes foot ulcer multi-disciplinary management, it could be argued that the current arrangements are inadequate to be considered satisfactorily publicly funded for seven diabetes-related foot recommendations [23,38].

An examination of all medications recommended for the management of diabetes-related complications suggests that all are publicly funded under the PBS system when prescribed by a medical or nurse endorsed prescriber [20-23,39,40]. Interestingly, many of those same recommended medications (particularly oral antibiotics for diabetes foot ulcer management) funded by PBS, are not covered by PBS when appropriately provided by an equivalent podiatry endorsed prescriber [39,40].

### How is additional government funding distributed to address different diabetes complication management?

In addition to PBS and MBS funding, the Federal Government in recent years has invested hundreds of millions of dollars into campaigns, programs, publications, research and/or additional resources (including capital and infrastructure) to manage the known complications of diabetes [41]. The distribution of this additional public funding for the management of diabetes complications appears to be more ad hoc, making it very difficult to make any direct comparison. However, a review of publicly available information from such sources as ministerial media releases, it appears that foot disease receives comparatively less support [41].

In reviewing the publicly available information, it is clear that the Australian Government has announced significant investment in the management of kidney, cardiovascular, and eye disease [41]. This includes in excess of $300 million to tackle kidney disease by funding the expansion of renal infrastructure, dialysis and support services across Australia and new drugs to combat kidney disease under the PBS [41]. Government has also allocated significant additional funding to combat cardiovascular disease, including up to $200 million for development and uptake of cardiovascular risk management tools and guidelines, investments in research, and establishment of a national centre for monitoring [41]. A number of cardiovascular disease initiatives have been federally funded through non-government organisations such as the Heart Foundation and the National Stroke Foundation [41]. A National Eye Framework has also been established with the responsibility for the funding of services to increase access to eye health and vision care [41]. Announcements to date include extra eye disease funding of up to $30 million, targeting increased consultation, training, equipment, surgical interventions and research to manage eye disease [41].

A search for similar Australian Government funding announcements over the last decade failed to identify any significant additional funding announcements specifically addressing foot or lower limb disease [41]. Furthermore, a cursory glance through the official Australian Institute of Health and Welfare (Australian Government funded) ‘diabetes related publications’ webpage seems to also highlight this lack of comparative government focus on foot disease [42]. Of the 55 publications listed most are generically diabetes titled, with five titles specifically related to diabetes cardiovascular disease, four to kidney disease and none address foot disease [42].

## Discussion

This paper identifies that the burden of diabetes-related foot disease faced by the Australian population is the second largest of the four main diabetes complications in terms of burden of disease and numbers of people affected. In contrast, it appears that government funding of national best-practice management recommendations, and any additional government funding programs, ranks the funding of diabetes-related foot disease management as a distant last compared to the other diabetes complications. A summary of these findings can be found in Table 3. Therefore, the public funding and resources afforded to improve outcomes related to diabetes-related foot disease burden seems to be disproportionately low when compared to the burden and funding attributed to other more well-known diabetes complications in Australia. This anomaly should be considered in the context of the apparent increases in diabetes-related amputation in Australia over the last decade.

**Table 3:** Summary of diabetes complication rankings in Australia

**Rank**	**Burden and cost of complication**			**Funding**	
	**Numbers affected**^**a**^	**Costs per episode**^**b**^	**Overall estimated costs**^**c**^	**Proportion guideline recommendations funded**^**d**^	**Additional programs funded**^**e**^
**First**	CVD	Kidney	Kidney	Kidney & CVD	Kidney
**Second**	Foot	Foot	CVD		CVD
**Third**	Eyes	Eyes	Foot	Eyes	Eyes
**Fourth**	Kidney	CVD	Eyes	Foot	Foot

a Numbers Affected: Mean% of the five parameters of High Risk and Acute complications in Table 1.

b Costs per Episode: Mean $cost of three ‘Total’ cost parameters in Table 2.

c Overall estimated costs: Mean $cost of three ‘Total’ cost parameters in Table 2 x Hospital admissions numbers in Table 1.

d Proportion guideline recommendations funded: Numbers of NHMRC endorsed diabetes complication guideline recommendations funded by MBS / Total number of NHMRC endorsed diabetes complication guideline recommendations.

e Additional Programs Funded: Additional total funding announcements of government in last 3 – 5 years.

Although it would appear that the Australian government has yet to specifically focus on reducing the burden of diabetes-related foot disease, there somewhat promisingly appears to have been an acceleration in the number of recent strategic publications released by national peak consumer, health professional and research bodies targeting the improvement in the prevention, assessment and management of diabetes-related foot disease [16,18,23,43]. Many of these documents cover the same territory in terms of recommendations to improve diabetes-related foot disease management and reduce the burden of diabetes-related foot ulceration and amputation in this country [16,18,23,43]. None, however, are yet to translate into public funding.

The NHMRC endorsed diabetes foot guidelines compiled by national diabetes research, clinical and consumer experts, under the auspices of the Baker IDI Heart & Diabetes Institute and George Institute, suggest that “a co-ordinated, national, multi-faceted, systems approach for implementation (of the guideline) is considered essential” [23]. It recommends as high priorities for implementation:

(i) funding and policy development to support timely access to health professionals and multi-disciplinary foot teams for people with high-risk and acute foot complications; in particular access to podiatrists, nurses and aboriginal health workers;

(ii) further training for allied health professionals to assess ulcer risk, wound debridement, appropriate wound dressing and pressure reduction, and;

(iii) electronic decision support tools for medical practitioners, allied health professionals and aboriginal health services [23].

The Australian Diabetes Society’s *Australian Diabetes Foot Network* (ADFN), a multi-disciplinary steering committee on diabetes-related foot disease, recently published a six point strategic plan to reduce Australian lower limb amputations in people with diabetes [16]. The plan, endorsed by the Australian Diabetes Society, Australasian Podiatry Council, Australian Diabetes Educators Association, Australian and New Zealand Society for Vascular Surgery, Australian Wound Management Association and Diabetes Australia, highlighted that the increase in diabetes-related foot disease in Australia “means a national focus on coordinated foot care is essential” [16]. Their six point strategic recommendations included:

(i) improved access to foot care via MBS for people with foot complications;

(ii) subsidies for evidence based treatments including pressure off-loading devices and medical grade footwear;

(iii) standardised national model for multi-disciplinary foot teams

(iv) national accreditation of multi-disciplinary foot clinics and health professionals;

(v) improving holistic diabetes care initiatives to “close the gap” on inequities in diabetes foot outcomes for indigenous Australians, and;

(vi) reporting of national incidence and outcomes of diabetes-related foot disease [16].

Interestingly, the previous Australian Diabetes Society (2000) [44] and national diabetes strategic plans (1998) [15], published over a decade ago, also included recommendations around routine podiatry care for people with high-risk feet, access to multidisciplinary team care for people with foot ulcers, increased access to foot care services for indigenous Australians, and a national diabetes foot care committee to oversee implementation of these national foot care activities and monitor outcomes [15,44]. The restatement of these same evidence-based strategies over 12 years on would seem to demonstrate the lack of systematic national implementation of these strategies and that existing arrangements have been insufficient in light of the increase in diabetes-related amputations over the same period. This lack of systematic investment seems to be in spite of the best efforts of the many multidisciplinary clinicians, health advocates and peak bodies.

In an attempt to provide practical implementable solutions within the constraints of the current MBS and PBS systems, the Australasian Podiatry Council [18] submitted a costed budget submission to the Australian Government for consideration in the 2012 Australian Budget round [18]. The plan practically addressed four of the points of the ADFN plan [16], including:

(i) implementing an annual national diabetes amputation rate report;

(ii) increasing access to podiatrists via MBS from a maximum of 5 visits to an average of 12 for those with diabetes-related foot complications and foot ulcers;

(iii) subsidising pressure off-loading devices for those with past or present foot ulcers; and

(iv) increasing access to MBS services for indigenous Australians with diabetes-related foot complications via indigenous health workers [18].

Subsequently, the peak national consumer body representing people with diabetes, Diabetes Australia, mirrored some of the recommendations of these plans in their recently released Diabetes National Election Agenda report (2012) [43]. The Diabetes Australia report addresses priorities for national implementation to improve the health of people living with diabetes, including:

(i) increasing access to MBS allied health visits from 5 – 12 for people with diabetes;

(ii) funding National Accredited Diabetes Centres (including multi-disciplinary teams), and;

(iii) providing all people with diabetes a comprehensive annual risk assessment [43].

This translates to up to ten national peak health professional, research and consumer bodies recommending similar strategies to reduce diabetes-related foot complications and amputation in this country [16,18,23,43]. These common strategies seem to include:

(i)  increasing access to podiatry, nursing, indigenous health workers and other allied health services via MBS;

(ii)  subsidising evidenced-based treatments such as pressure off-loading devices via government funding;

(iii)  implementing standard models of multi-disciplinary foot care teams across the nation;

(iv)  reporting and monitoring of annual national diabetes foot disease rates, and;

(v)  urging the increased uptake of evidence-based guidelines [16,18,23,43].

These common strategies when implemented in other countries appear to significantly reduce diabetes-related amputation rates and costs [45-54]. Many other developed nations systematically implementing such evidence-based strategies display amputation rates approximately half that of Australia [12,18,33,34]. The UK has reported amputation rates as low as seven per 100,000 population [50,53] or nearly one third of the rate faced in Australia [17,18,34]. Furthermore, an international cost-utility analysis study concluded that just a 25% reduction in ulceration and amputation rates by implementing and funding best-practice systems to manage diabetes foot complications is a ‘cost-saving strategy’ [55]. The Australasian Podiatry Council plan suggested that with the implementation of such strategies and assuming similar outcomes to those found in other nations, that an overall reduction in amputations and hospitalisation of between 24 to 90% was achievable in the Australian context [18]. This reduction translates to an estimated overall cost saving to Australia of $220 to 400 million annually [18]. Thus, it would appear Australia could learn many lessons from other comparable nations that have invested in these well-known evidence-based strategies, which have reaped the associated benefits of reduced amputation, hospitalisation and cost for diabetes-related foot disease.

Overall, the burden of diabetes-related foot disease in Australia is high and funding evidence-based diabetes-related foot management seems to significantly reduce this burden when implemented in other comparable nations. This simple yet very effective strategy, of funding evidence-based recommendations, also appears to have enjoyed success in other Australian diabetes complications in terms of reducing devastating diabetes complication outcomes in Australia. It would therefore seem intuitive to follow this same strategy and ensure that at a minimum all national best-practice guideline recommendations on the management of diabetes complications are publicly funded to reduce the significant associated morbidity and mortality faced in Australia and improve the limbs and lives of Australians with diabetes.

## Conclusions

Diabetes has been rated as one of the greatest public health challenges to face Australia. This paper has demonstrated that diabetes-related foot complications, in comparison to other diabetes complications, make up a considerable portion of the diabetes-related burden of disease in Australia. In contrast to other diabetes complications, it appears that a very low number of national best-practice recommendations have been incorporated into publicly funded models of care, and that foot disease lacks any additional public funding. These conspicuous gaps may significantly contribute to the poor outcomes of diabetes-related foot complications facing Australia in comparison to other Australian diabetes complications and international diabetes-related foot disease outcomes. In the spirit of unanimous UN action on diabetes, the promising diabetes-related foot results seen in other nations, and the constant urging of multiple diverse national peak bodies, it seems like “its time” to equally invest in diabetes-related foot disease in Australia.

## Competing interests

PAL and JMG were members of the Australian Diabetes Foot Network and along with SMB co-authored the *Medical Journal of Australia* article cited in this manuscript. PAL, JRR and AS are board members of the Australasian Podiatry Council and were involved in the drafting of the Australasian Podiatry Council Budget Submission cited in this manuscript. PAL was a member of the guidelines advisory committee that oversaw the development of the NHMRC diabetes foot guideline cited in this manuscript. The Australasian Podiatry Council co-funds the *Journal of Foot and Ankle Research*.

## Authors’ contributions

PAL conceived, designed, searched literature, contributed to discussion, wrote and reviewed/edited the manuscript. JMG searched literature, contributed to discussion, wrote and reviewed/edited the manuscript. JRR, AS, SMB searched literature, contributed to discussion and reviewed/edited the manuscript. All authors read and approved the final manuscript.
